# Supplementary material for: CoVigator—A Knowledge Base for Navigating SARS-CoV-2 Genomic Variants
Source: Viruses. 2023 Jun 17;15(6):1391. doi: 10.3390/v15061391 (PMC10304279; doi:10.3390/v15061391)
Supplement: Supplementary file 1 [file viruses-15-01391-s001.zip › viruses-2451791-supplementary.pdf]

Supplementary Material

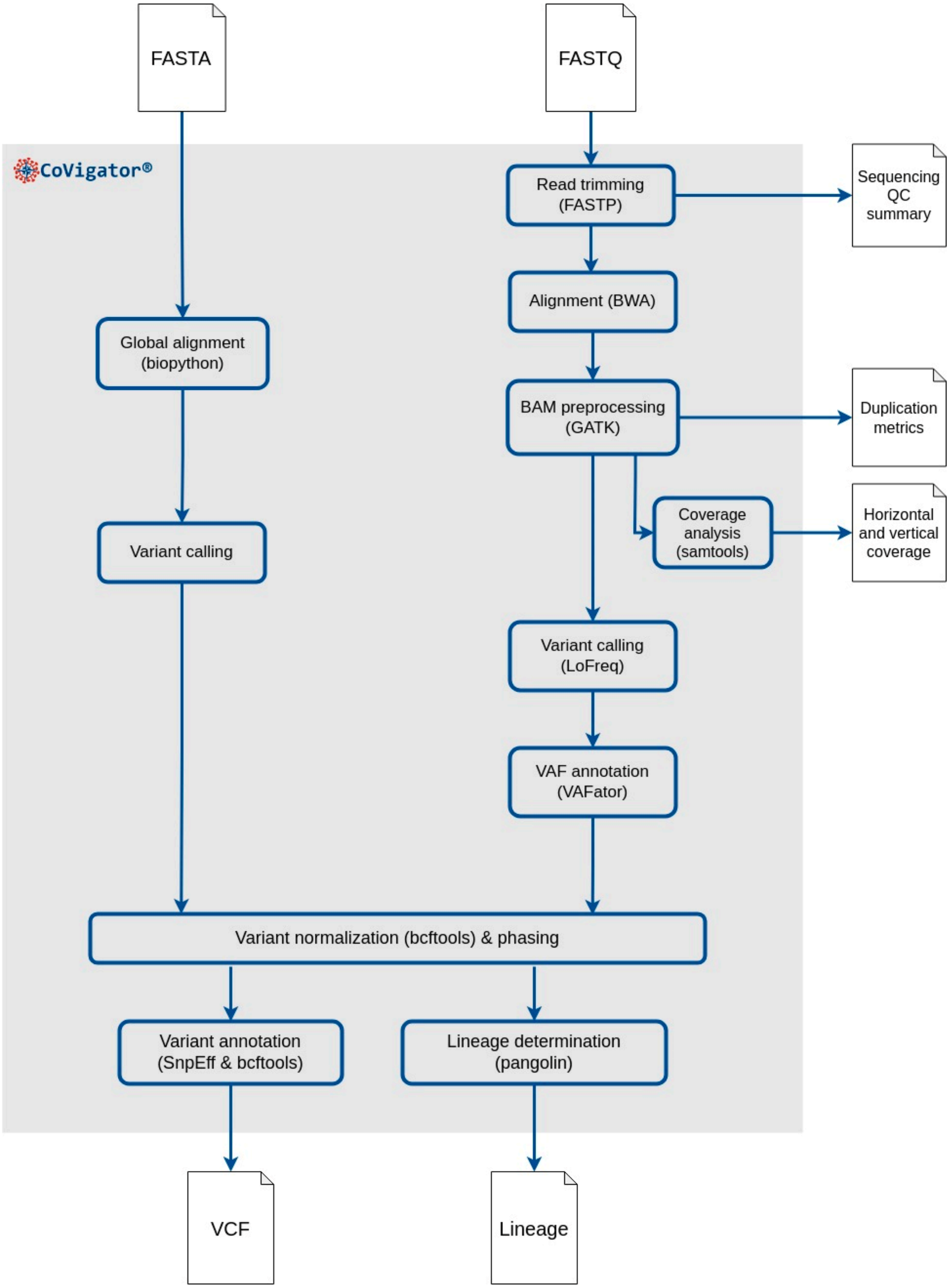

Figure S1. Workflow of CoVigator pipeline. The input can be either FASTQ or FASTA files. The output are multiple VCF files (one per variant caller), a Pangolin lineage file, horizontal and vertical coverage results and the FASTP sequencing summary. Optionally, the BAM file and other intermediate files may be kept in the output.

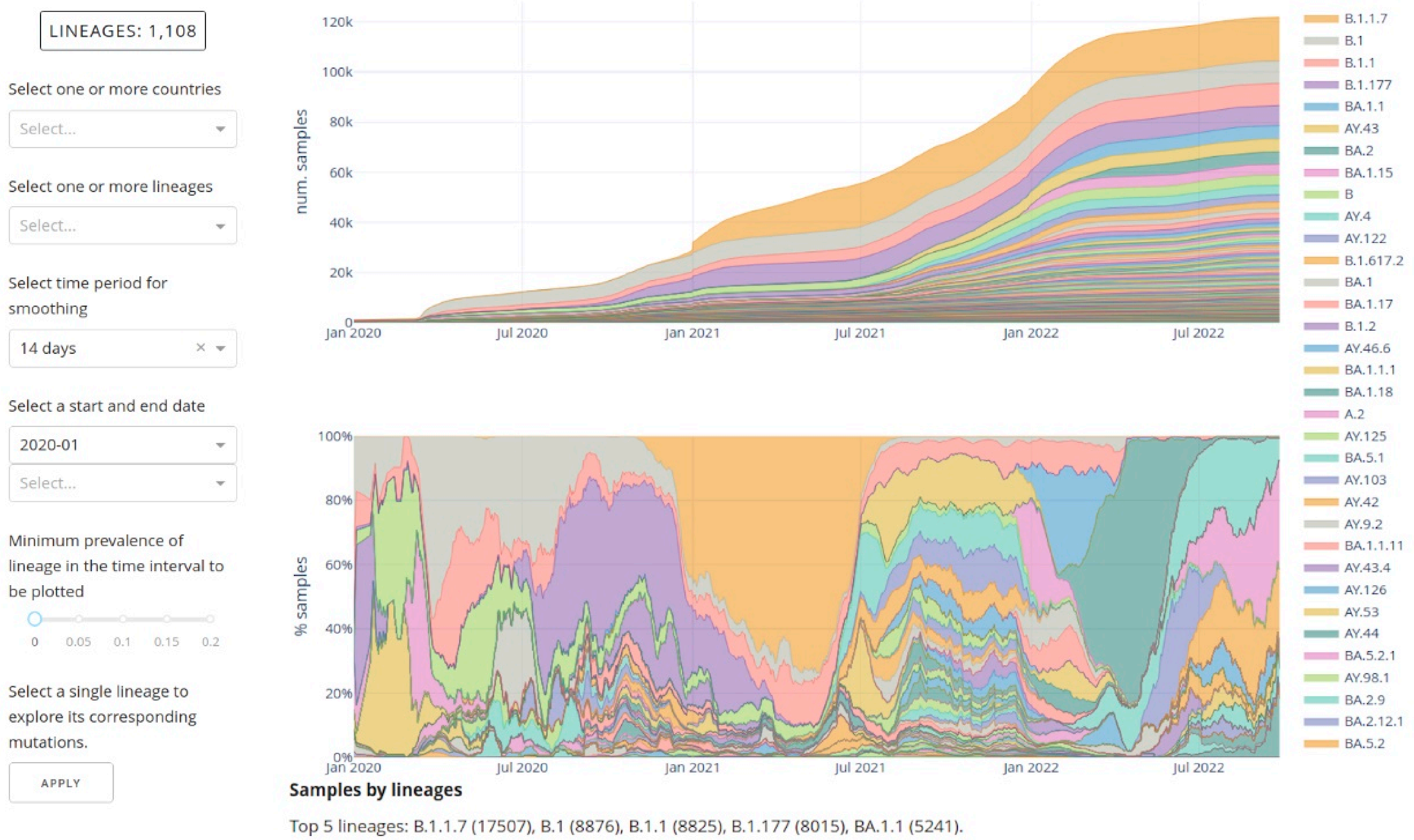

Figure S2. Screenshot of the lineages tab in the ENA dataset. On the left the filters for countries, lineages and time range; and plot settings for smoothing and minimum prevalence of a lineage. On the top right the accumulation through time of samples by lineage. On the bottom right the percentage of samples by lineage.

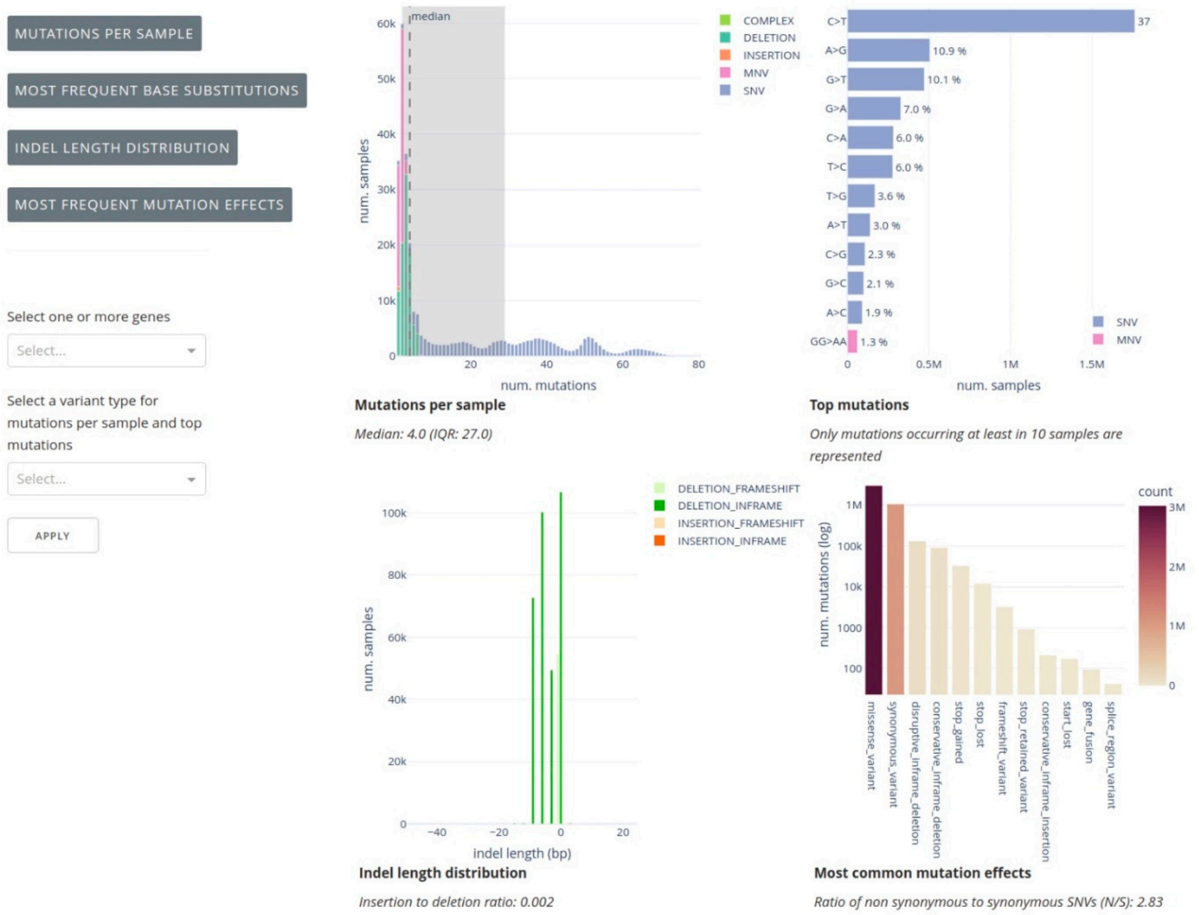

Figure S3. Screenshot of mutation statistics tab in the ENA dataset. On the left filters for genes and variant types. On the top left the distribution of the number of mutations per sample. On the top right the most frequent substitutions. On the bottom left the indels length distribution. On the bottom right the distribution of the different types of mutations.

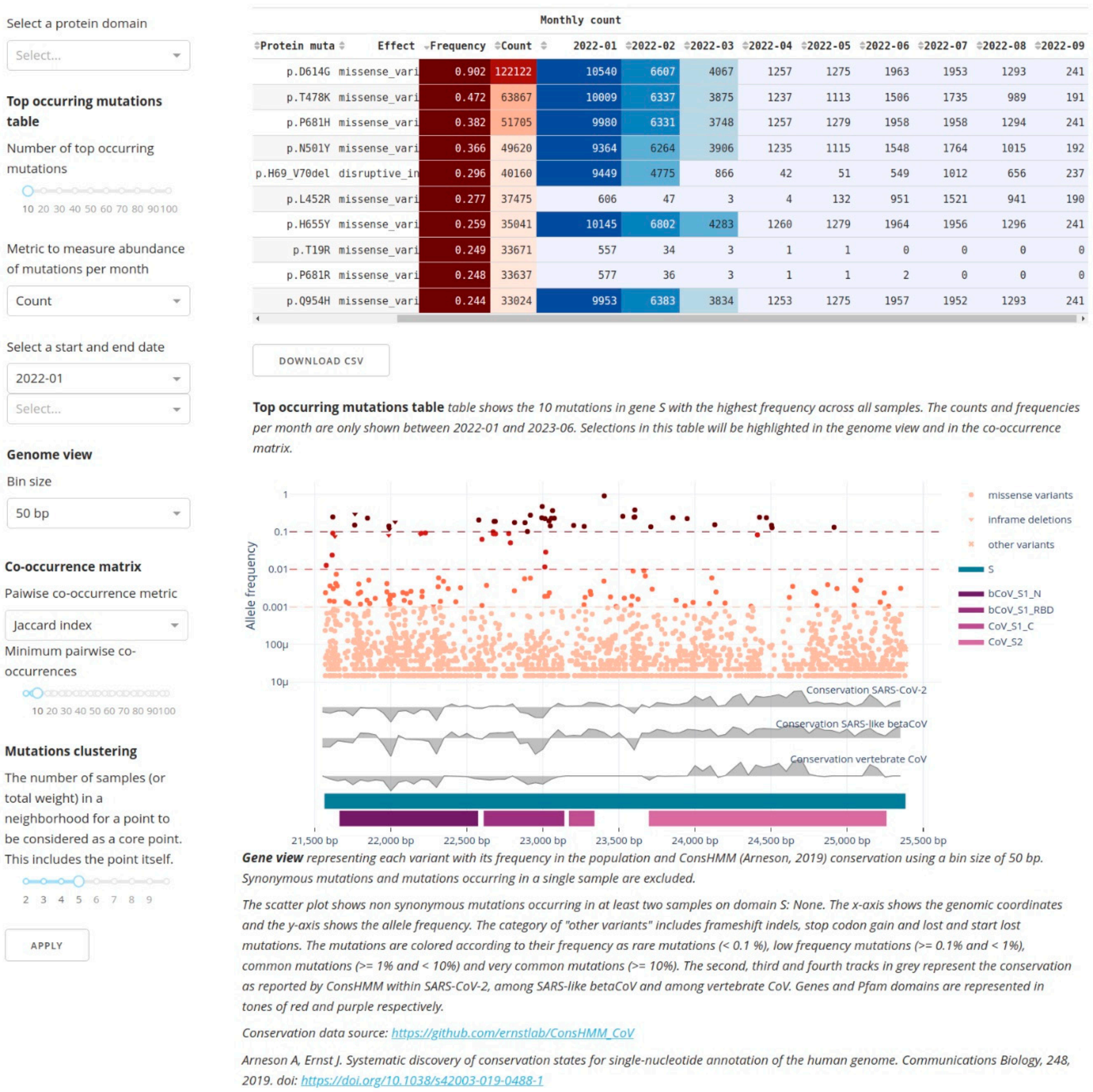

Figure S4. The recurrent mutations tab for the spike protein in the ENA dataset. On the left the filters gene, protein domain, number of top occurring mutations, abundance of mutation metric, date range, bin size for the genome view, pairwise co-occurrence metric, minimum pairwise co-occurrences and the minimum number of samples in the neighborhood to consider a mutation a core point during clustering. On the top, right the top ten occurring mutations in the spike protein with the number of observations per month in the defined date range. On the bottom left a view of the spike protein including mutations by their frequency in the ENA dataset, conservation metrics and protein domains.

Protein muta	Effect	Frequency	Count	2022-01	2022-02	2022-03	2022-04	2022-05	2022-06	2022-07	2022-08	2022-09
p.D614G	missense_vari	0.995	6078899	629317	432355	438232	192742	151582	153189	159935	112020	67847
p.T478K	missense_vari	0.807	4928723	579407	410405	422336	183123	146936	149648	157062	110471	67469
p.P681H	missense_vari	0.515	3146876	604435	430862	436851	192143	151388	152755	159858	112031	67753
p.G142D	missense_vari	0.506	3088413	47747	126942	355205	183727	148710	151631	158903	111078	67514
p.N501Y	missense_vari	0.494	3018055	556780	409913	423024	183476	147276	150238	158035	111076	67596
p.L452R	missense_vari	0.473	2888865	18319	632	236	543	8750	74081	138212	106280	65145
p.H655Y	missense_vari	0.435	2659663	611453	431604	437945	192753	151621	153165	159944	111956	67823
p.N969K	missense_vari	0.431	2631195	612150	432279	437819	192942	152099	153252	160133	112195	67752
p.N679K	missense_vari	0.429	2620336	604341	431354	437804	192687	151481	152893	159944	112072	67782
p.D796Y	missense_vari	0.429	2617320	605491	429548	436659	192384	151369	152695	159601	111899	67652
p.Q954H	missense_vari	0.429	2619188	606459	428592	437504	192803	151997	152978	159878	112071	67682
p.S477N	missense_vari	0.414	2531392	560236	409559	421887	183047	146803	149543	156761	110195	67403
p.G339D	missense_vari	0.411	2507869	567127	415742	422145	186828	146787	147075	154758	108621	65334
p.E484A	missense_vari	0.409	2497845	561527	410237	421800	183144	147054	149911	157569	110700	67395
p.Y505H	missense_vari	0.408	2491128	555941	409835	423082	183674	146700	149821	157713	110884	67517
p.Q498R	missense_vari	0.408	2489415	554610	408290	422300	183312	147214	150181	157957	111049	67561
p.P681R	missense_vari	0.405	2472591	17053	585	168	26	32	103	71	12	12
p.N764K	missense_vari	0.401	2446213	548379	417723	428889	187972	146841	147512	155797	110051	66892
p.S375F	missense_vari	0.401	2446415	538091	403848	419029	184838	146624	146416	154453	108927	66390
p.S373P	missense_vari	0.401	2446306	537717	404219	419048	185173	146530	146413	154384	108921	66396

DOWNLOAD CSV

Top occurring mutations table *table shows the 20 mutations in gene S with the highest frequency across all samples. The counts and frequencies per month are only shown between 2022-01 and 2023-06. Selections in this table will be highlighted in the genome view and in the co-occurrence matrix.*

Figure S5. Top 20 mutations in the spike protein from COVID-19 Data Portal. The month ranges are configurable from the dashboard and the raw data in CSV format is downloadable.

Supplementary Method for co-occurrence analysis

The co-occurrence analysis employs the similarity matrix with Cohen's kappa coefficient between every pair of variants.

$$Jaccard\ similarity\ (J) = \frac{a \cap b}{a \cup b}$$

$$Cohen's\ kappa\ coefficient = \frac{1 - J}{1 - \ e^{-a \cap b}}$$

Where a and b are the count of observations of two variants. The intersection of a and b are the co-occurring observations of both variants and the union of a and b are the count of samples on where any of the two variants were observed without repetitions.

Clustering is performed using the OPTICS algorithm [69] as implemented in scikit-learn library version 0.24.2 [70] with max_eps=1.4 and min_samples=5, although the latter can be parametrized in the dashboard. Other parameters are set to default. Even though in the dashboard the heatmap can show different similarity metrics the clustering is always performed with Cohen's kappa coefficient.

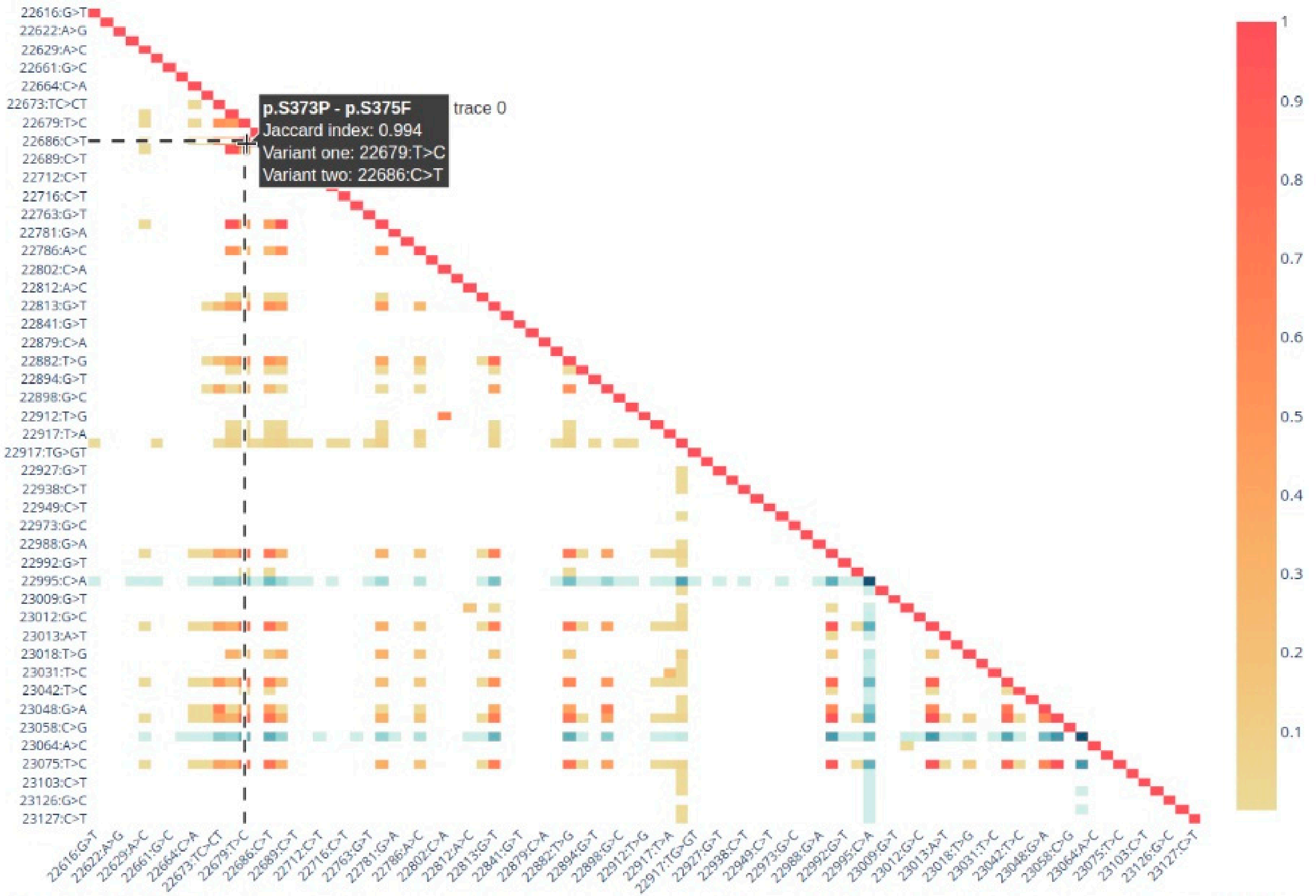

Co-occurrence matrix *showing variant pairs co-occurring in at least 10 samples (this value is configurable). The metric in the co-occurrence matrix can be chosen among counts, frequencies, Jaccard index or Cohen's kappa coefficient. The Cohen's kappa coefficient introduces a correction to the Jaccard index for mutations with low occurrence. The diagonal contains the total counts or just 1.0 in the other metrics. The upper diagonal is not shown for clarity. Synonymous mutations are excluded. Different genomic mutations causing the same protein variant are not grouped.*

Figure S6. Jaccard index co-occurrence matrix on the Receptor Binding Domain of the spike protein

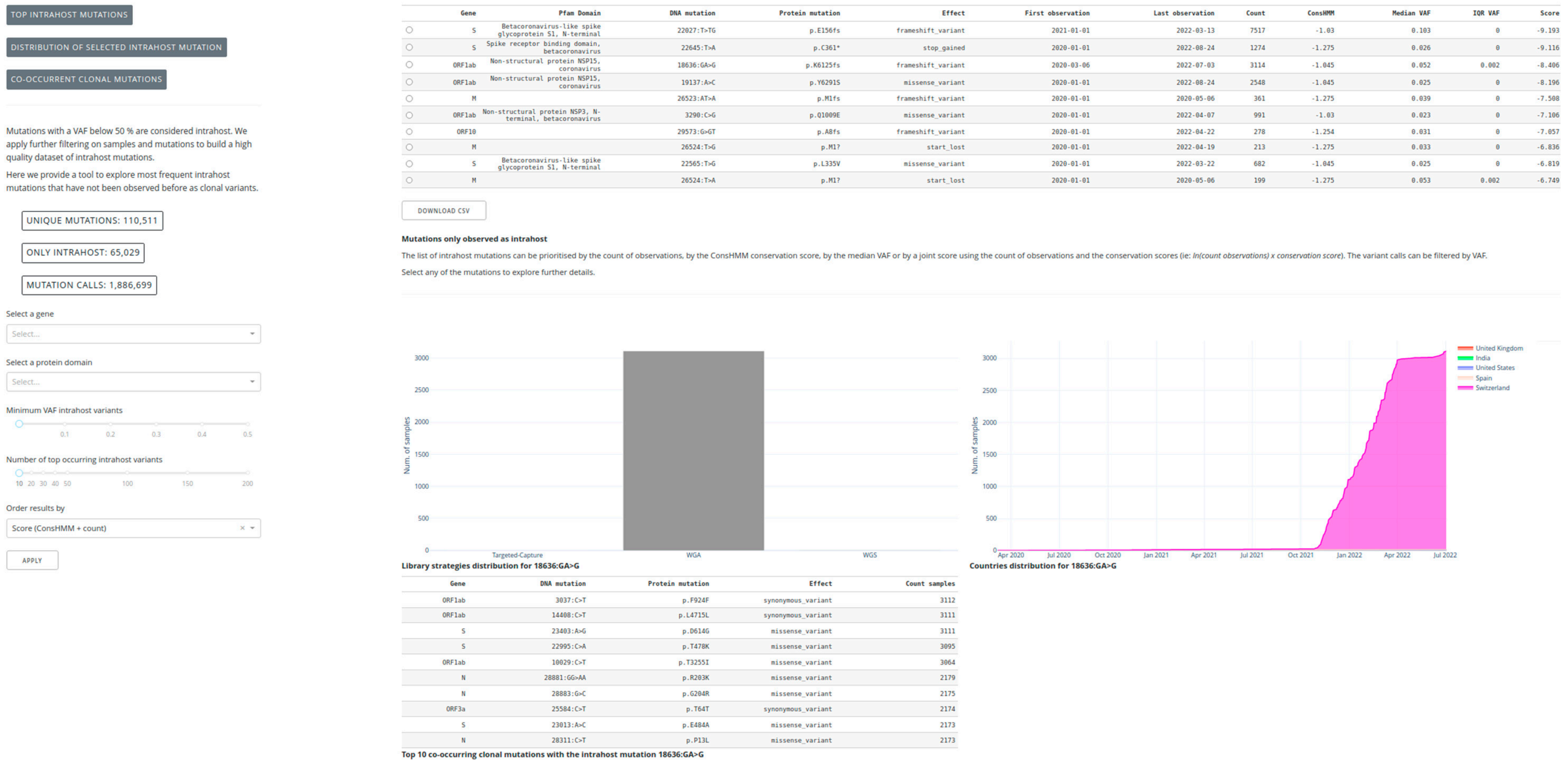

Figure S7. The intrahost mutations tab helps to prioritize recurrent intrahost mutations that undetected before in clonal state. When selecting a particular intrahost mutation it shows the distribution of this mutation over time, geography and different library strategies. Also, identifies the clonal mutations with which it co-occur

Supplementary Method for extension to other viruses

CoVigator was developed specifically for SARS-CoV-2 motivated by the COVID-19 pandemic; but its methodology is extensible to similar infectious organisms. In particular the CoVigator pipeline should provide sound results for either DNA or RNA viruses, single or double stranded that are not segmented. In the case of double stranded viruses the phasing algorithm may have limitations for overlapping genes. Also, the pangolin lineage determination does not work for other viruses than SARS-CoV-2.

We have in particular used the CoVigator pipeline to analyze two other viruses: Chikungunya (CHIK) (unpublished data) and Ebolavirus (EBOV) (492 public Illumina samples from ENA downloaded on 14/06/2023), both single-stranded RNA viruses.

The CoVigator accessor that downloads metadata and FASTQ files from the ENA API can be used given any taxonomy identifier, e.g.: "covigator-accessor --tax-id 186538". The taxonomy identifier can be retrieved from the EBI taxonomy REST API given a scientific name, e.g.: https://www.ebi.ac.uk/ena/taxonomy/rest/scientific-name/Zaire%20ebolavirus. Finally the pipeline can be used with any arbitrary reference as described in the documentation https://covigator.readthedocs.io/en/latest/03_pipeline.html#using-a-custom-reference-genome. We foresee a further adaptation of the CoVigator processor and dashboard to fully support other viruses.
